# NMR Metabolomics and Chemometrics of Lettuce, *Lactuca sativa* L., under Different Foliar Organic Fertilization Treatments

**DOI:** 10.3390/plants11162164

**Published:** 2022-08-20

**Authors:** Virginia Lanzotti, Attilio Anzano, Laura Grauso, Maurizio Zotti, Adriana Sacco, Mauro Senatore, Mauro Moreno, Marcello Diano, Maddalena Parente, Serena Esposito, Pasquale Termolino, Emanuela Palomba, Astolfo Zoina, Stefano Mazzoleni

**Affiliations:** 1 Dipartimento di Agraria, Università di Napoli Federico II, Via Università 100, 80055 Portici, Italy; 2 Institute for Sustainable Plant Protection (IPSP), National Research Council of Italy (CNR), 80055 Portici, Italy; 3 M2M Engineering sas, Business Innovation Center, Science Center, Via Coroglio, 80124 Naples, Italy; 4 Institute of Biosciences and Bioresources (IBBR), National Research Council of Italy (CNR), 80055 Portici, Italy

**Keywords:** plant spectral information, natural product chemistry, food plant, NMR spectroscopy, *Fusarium oxysporum*, *Artrospira platensis*

## Abstract

Lettuce plants were grown in a greenhouse affected by the fungal pathogen *Fusarium oxysporum* to test the effects on plant metabolomics by different organic treatments. Three foliar application treatments were applied: a commercial compost tea made of aerobically fermented plant organic matter, a pure lyophilized microalga *Artrospira platensis*, commonly named spirulina, and the same microalga previously exposed during its culture to a natural uptake from medium enriched with *F. oxysporum* fragmented DNA (NAT). The experiment is the first attempt to observe in field conditions, the use and effects of a natural microbial library as a carrier of pathogenic fungal DNA for disease control. Untargeted NMR metabolomics and chemometrics showed that foliar organic application significantly reduced fumaric and formic acids, aromatic amino acids, and nucleosides, while increasing ethanolamine. A strong decrease in phenolic acids and an increase in citric acid and glutamine were specifically observed in the NAT treatment. It is noteworthy that the exposure of a known biostimulant microalga to fungal DNA in its culture medium was sufficient to induce detectable changes in the metabolomic profiles of the fertilized plants. These findings deserve further investigation to assess the potential relevance of the presented approach in the field of crop biostimulation and biocontrol of plant pathogens.

## 1. Introduction

In the last decade, metabolomic approaches have been used as a tool to understand the impact of environmental stresses by monitoring the qualitative and quantitative change of metabolites extracted either from whole plants or single tissues [1]. ^1^H NMR metabolomics is one of the most used approaches, as it provides a fast and wide overview of the metabolite content of plant extracts with a relatively easy and fast sample preparation [2]. The outcome of the metabolomic analysis is a “fingerprint” that gives information about the current growing condition of the plant. Several studies tried to describe the metabolite changes resulting from stress conditions, and it seems that different stresses cause either an increase or decrease in specific metabolites [3].

Lettuce, *Lactuca sativa* L., is a well-known food plant worldwide due to its use in both fresh salads and cooked soups. It is a member of the Asteraceae family, and it was first described in 1753 by Carl Linnaeus in the second volume of his *Species Plantarum*. The whole plant is rich in a milky sap that flows out from any wounds of the basal stem [4]. Lettuce has also been reported in folk medicine for several therapeutic uses such as a carminative, diuretic, emollient, febrifuge, hypoglycemic, hypnotic, parasiticide and sedative [5]. Lettuce is generally cultivated as an annual crop, requiring relatively low temperatures to prevent it from early flowering. It can suffer from numerous nutrient deficiencies, as well as be plagued by several insects and pests, fungal and bacterial diseases [6].

Due to the importance of lettuce as a food crop, different studies have been done on this plant species using metabolomic approaches. The study published by Sobolev et al. [7], was based on a combination of 1D and 2D NMR approaches, and provides a detailed characterization of *L. sativa* polar and non-polar extracts.

More recently, the paper by Yang et al. [8] characterized the metabolome of 30 different lettuce cultivars, with large genetic diversity with provenances from different continents, including America, Asia, and Europe. The reported data allowed differentiation of the analyzed plants, showing different compositions of secondary metabolites. In the same year, Van Treuren et al. [9] performed an extensive metabolomic analysis on 150 different accessions. The identification of many metabolites, through a LC-MS approach, resulted in a clear cluster structure, separating all the cultivated lettuce genotypes from another group of the wild ones. Main differences were found in the relative abundance of chlorogenic, chicoric and caftaric acids, reported to be significantly higher in the wild types.

Several works focused on the effects of specific biotic and abiotic stresses on lettuce leaf extracts. Zhou et al. [10] found that exposure of *L. sativa* to low nitrogen treatment increased the accumulation of phenolics, sugars, and organic acids (citric and isocitric acid). They also found a decreasing trend for malic and fumaric acids. A mixed response has been found for amino acids with an increasing or decreasing trend for each compound compared to the control. Higher levels of flavonoids and of three amino acids (valine, isoleucine and threonine) were also detected in *L. sativa* leaves by Wei et al. [11] in plants under thermal stress. Another recent study by Matamoros et al. [12] reported on the effects of different fertilization treatments on the lettuce metabolome using LC-MS. Five different treatments were compared, including sewage sludge, swine manure, chemical fertilization, mixed organic fraction of municipal solid waste (compost derived from parks, gardens, and kitchen wastes), and a control with no fertilization. Sewage sludge and chemical fertilization resulted in higher levels of amino acids, probably due to higher nitrogen supply. Conversely, treatments with swine manure, the organic fraction of municipal solid waste, and control, corresponding to lower nutrient levels, led to a higher organic acids content, and increased levels of many sugars.

The need for reduction in chemical pesticides in modern agriculture is strongly increasing the interest in organic based products such as compost from different source materials, also because these compounds have been reported to have suppressive effects on different plant pathogens, especially fungal diseases [13,14,15,16,17], but also nematodes [18]. The idea of pathogen control finds correspondence in traditional practices [19] and specific applications have been reported to fight fungal pathogens, such as *Phytium*, *Rhizoctonia* and *Sclerotinia* [20]. Additionally, the control of *Fusarium* wilt has been attempted in flower plants [21] and tomato, coupling beneficial *Trichoderma* in combination with sheep manure [22]. Moreover, there is a strong interest for organic materials from all sorts of different natural sources, including sea weeds [23], and microalgae [24,25], as well as fish emulsions [26], with reported biostimulant effects when applied in either foliar or seed treatments. Among microalgae, a renowned species is *Artrospira platensis* commonly named spirulina which is widely cultivated and used as biostimulant for both plant fertilizers and animal food integrator products.

It is known that plants react to abiotic and biotic stresses with different metabolic responses [3]. In particular, when infected by pathogens plants change their metabolic profile. Here, we present the results of a first study aiming to assess the effects of organic foliar treatment on the metabolic profiles of common lettuce in a field characterized by high infestation levels of the fungal pathogen *Fusarium oxysporum lactucae* (FOL). Untreated lettuce was used as control plant. Three different treatments were designed: a first with a commercial organic plant material mixture used for the preparation of a so-called compost-tea (CT), i.e., a supernatant of an aerobic fermentation of such an organic substrate; a second with *Artrospira platensis* (spirulina—SP), as biofertilizer and a biostimulant product [24,25]; and a third with the same *A. platensis* previously exposed to a natural uptake of FOL DNA (NAT), to test the idea of self-DNA inhibition as proposed by Mazzoleni et al. [27,28].

Three questions were addressed as aims of this work: (i) is foliar organic fertilization reducing the occurrence of lettuce disease by FOL? (ii) is lyophilized *A. platensis* (spirulina) added to foliar applications, producing comparable effects to the use of generic compost-tea made of mixed plant organic matter? (iii) does *A. platensis*, when exposed to pathogen DNA, show changes in its biostimulant effect on lettuce infected by FOL?

## 2. Results

### 2.1. Plant Analysis

**Figure 1:**
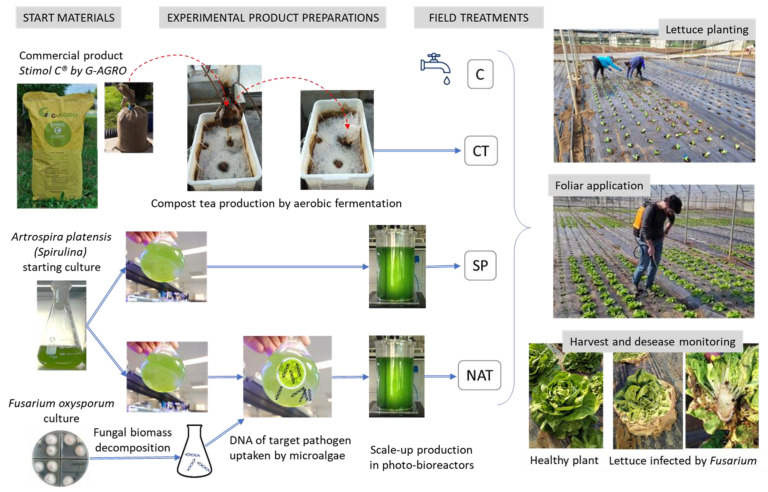
Schematic description of the experimental design of *Lactuca sativa* organic fertilization. Control plots were irrigated with pure water. Treatments were done by foliar application with a water solution of commercial compost tea (CT), spirulina (SP), and spirulina previously exposed to natural uptake of *Fusarium* DNA (NAT).

Figure 1 schematically describes the experimental setting of the work. Control plants (C) were supplied with only pure water. Compost tea (CT) treatment used as starting material a commercial product Stimol C^®^ by G-AGRO. The product solubilized in water was aerobically fermented to give the final fertilization solutions used for plants. Lettuce was hand planted in parcels.

**Figure 2:**
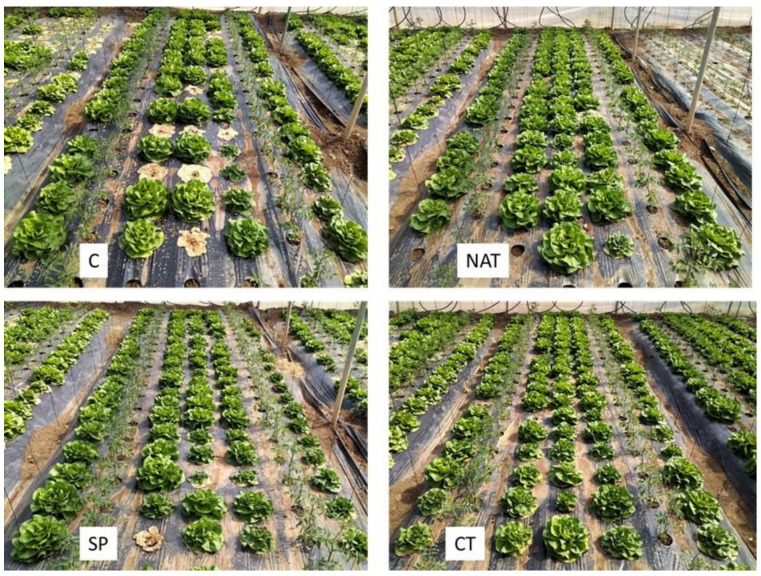
Examples of experimental plots of *Lactuca sativa* organic fertilization tests: Control (C) irrigated only with water and showing higher occurrence of *Fusarium oxysporum* attack. Treatments by foliar application with water with added spirulina (SP), spirulina after natural uptake of *F. oxysporum* DNA (NAT), and commercial compost tea (CT).

The organic fertilization produced in the observed experimental conditions some beneficial effects on lettuce growth and disease control (Figure 2). In particular, the average number of plants killed by *F. oxysporum* infection was found to be significantly higher in the control plants compared to the treated plants (Table 1). Some beneficial effect, though less significant, was also observed in terms of the number of stunting plants (smaller sized with some desiccation of basal leaves, reflecting relatively milder levels of fungal infection. Among the organic treatments, the NAT, i.e., the microalga exposed to the presence of *Fusarium* DNA, showed a better performance compared to the pure spirulina and to the compost tea (Table 1 and Figure 2).

### 2.2. Metabolomic Analysis

**Figure 3:**
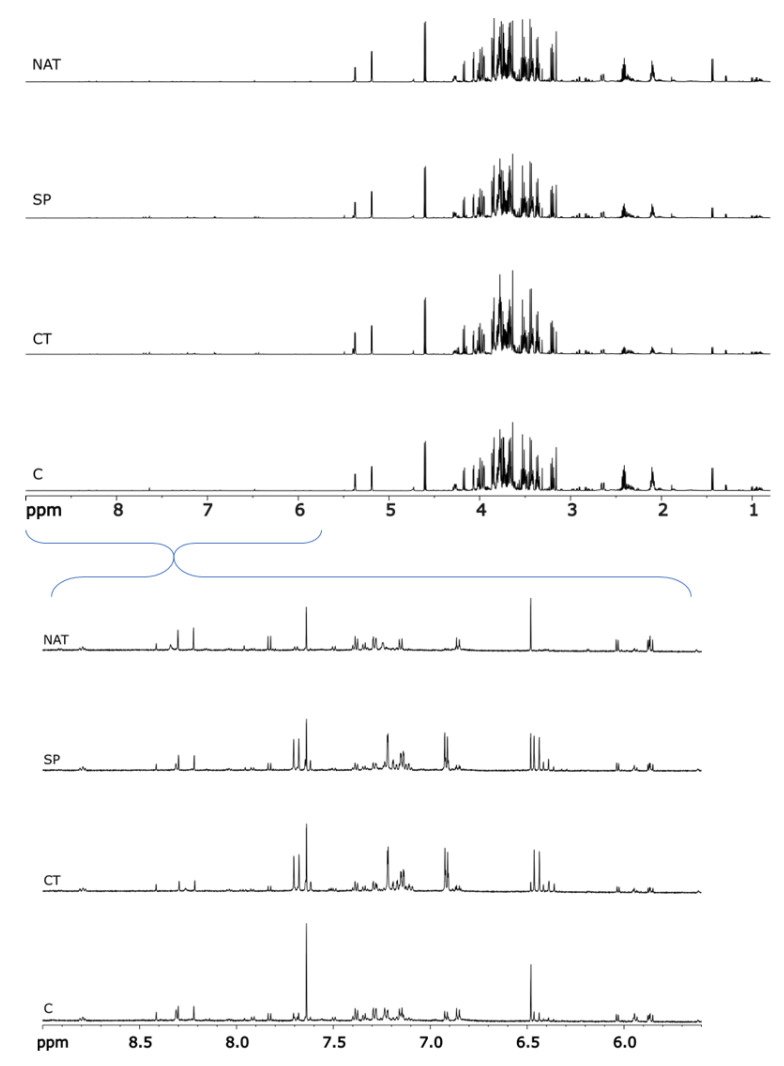
Full ^1^H NMR spectra in deuterium oxide (D_2_O) at 600 MHz of *L. sativa* leaf extracts of the control plant (C) and the three treatments (CT, SP and NAT). Only one of the three replicates is shown for each treatment (**Top**), all replicates are reported in Figure S1. Expanded high field region of the ^1^H NMR spectra (**Bottom**).

Twenty grams of lettuce leaves afforded, after lyophilization, an average of 1.12 g of dried lettuce powder, resulting in an average moisture content of 94.41%. Samples obtained from the different treatments were analyzed in triplicate and the obtained organic extracts subjected to NMR metabolomics (Figure S1). The NMR data obtained (Figure 3, full spectra) showed no significant change among treatments and control plants from a qualitative point of view. However, comparison of the spectra evidenced changes in the metabolite composition (Figure 3, expanded low field region).

Therefore, we analyzed the spectra peak by peak to identify the main metabolites. Spectral identification was performed with the aid of the 2D NMR spectra acquired, which included COSY, HSQC and HMBC (Figures S2–S4) and by comparison with the data for standard compounds available in the laboratory and reported in the literature. This allowed identification of single metabolites in the extract mixture. Table 2 lists the metabolites found using ^1^H NMR analysis in the analyzed extracts. Compounds included primary metabolites such as carbohydrates, amino acids, nucleosides, and secondary metabolites such as phenolic acids.

**Figure 4:**
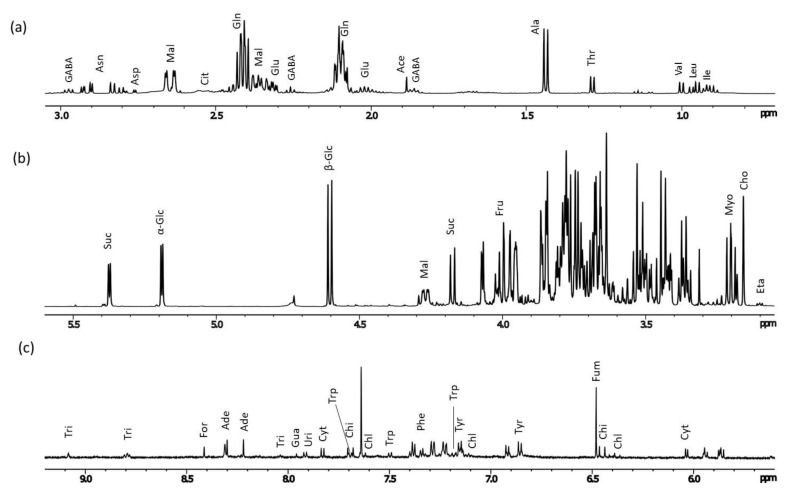
^1^H NMR spectra in deuterium oxide (D_2_O) at 600 MHz of *L. sativa* leaves extracts (control plant) showing the identified metabolites: (**a**) spectral region from 0.7 to 3.0 ppm; (**b**) spectral region from 3.1 to 5.5 ppm; (**c**) spectral region from 5.6 to 9.5 ppm vertically expanded.

The high field region of the spectra contains the methyl groups of the branched amino acids (Figure 4a). Specifically, two doublets at δ 0.95 (*J* = 7.0 Hz) and δ 1.00 (*J* = 7.0 Hz) were diagnostic in determining leucine (Leu) and valine (Val), respectively. Isoleucine (Ile) was assigned through a triplet at δ 0.91 (*J* = 7.0 Hz) (Table 2). Threonine (Thr) was assigned through a doublet at δ 1.29 (*J* = 7.0 Hz) because of its characteristic β-CH_3_. Differently from the other aliphatic amino acids, the β-CH_3_ group of alanine (Ala) resonated as a doublet at δ 1.43 (*J* = 7.0 Hz) due to its proximity to the nitrogen atom. Asparagine (Asn) was identified by the β-CH signals resonating at δ 2.86 (dd, *J* = 17.4 and 3.8 Hz) and 2.87 (dd, *J* = 16.9 and 7.3 Hz). The double doublet at δ 2.76 (*J* = 17.4 and 3.8 Hz) was assigned to the β-CH_2_ of aspartic acid (Asp). Then, glutamic acid (Glu) and glutamine (Gln) were detected based on β-CH_2_ and γ-CH_2_ resonating respectively at δ 2.02 (m) and 2.32 (m, *J* = 7.0 Hz) for Glu and δ 2.10 (m) and 2.43 (m) for Gln. Characteristic signals for γ-aminobutyrate (GABA) were found at δ 2.26 (t, *J* = 7 Hz, α-CH_2_), 1.87 (m, β-CH_2_), and 2.97 (t, *J* = 7 Hz, γ-CH_2_). Aromatic amino acids were identified in the low field region of the ^1^H NMR spectra from δ 6.83 to 7.70 (Figure 4c) and identified as phenylalanine (Phe), tyrosine (Tyr) and tryptophan (Trp) (see Table 2).

Among the organic acids, signal characteristics for five were detected across all spectral regions (Figure 4a–c and Table 2). At high field regions, the α-CH_3_ group of acetic acid (Ac) was found at δ 1.88 (s). In the same region citric acid (Cit) was identified by its CH_2_ group whose protons resonated as a double doublet at δ 2.59 (*J* = 15.0, 15.0 Hz). Malic acid (Mal) was identified by the following characteristic signals: δ 2.36 (dd, *J* = 15.7 and 8.9 Hz, β’-CH_2_), 2.65 (dd, *J* = 15.7 and 3.7 Hz, β-CH), 4.28 (dd, *J* = 8.9 and 3.7 Hz, α-CH). Finally, in the aromatic region, the acid proton formic acid (For) was identified at δ 8.41 (s, COOH) while a proton characteristic for fumaric acid (Fum) resonated at δ 6.48 (s, α-CH).

In the spectra of lettuce aqueous extracts, overlapping signals for sugar protons were observed from 3.33 to 3.87 ppm, which is a very crowded region of the ^1^H NMR spectra (Figure 4b). Outside this region, the diagnostic anomeric proton signal (H1) of α-glucose (α -Glc) was identified at 5.19 (d, *J* = 4.0 Hz), while the anomeric proton of β-glucose (β -Glc) was found at δ 4.61 (d, *J* = 8.0 Hz) (Table 2 and Figure 4b). Signals identifying fructose (Fru) were found at δ 3.90 (CH-4) and 4.00 (CH_2_-6). Sucrose (Suc) was identified by the anomeric proton of the glucose moiety resonating at δ 5.38 (d, *J* = 4.0 Hz). Lastly, the sugar alcohol myo-inositol (Myo) showed a peak at δ 3.20 corresponding to H5. The identification and spectral assignments of the reported sugars (Table 2) were obtained both by comparison with NMR data (chemical shifts and coupling constants) of standard sugars [29] and by analysis of 2D NMR spectra (Figures S2–S4).

Nucleosides were identified in the low field region of the spectra, by their characteristic resonances of the aromatic protons of the heterocyclic rings (Figure 4c and Table 2). Thus, the purine nucleoside adenosine (Ade) showed two singlets at δ 8.21(CH-2) and 8.40 (CH-8) for the nitrogen base and a doublet at δ 6.03 (d, *J* = 3.5 Hz) due to the anomeric proton of the ribose moiety. The purine nucleoside guanosine (Gua) was identified by the typical singlet at δ 7.95 (CH-8). The pyrimidine nucleoside Cytidine (Cyt) showed a characteristic doublet at δ 7.82 (*J* = 7.0 Hz) assigned to CH-6, while the same signal for uridine (Uri) resonated at δ 7.91 (*J* = 7.0 Hz, CH-6).

In addition to the polar compounds described above, ^1^H NMR spectra from the polar extracts of lettuce leaves indicated the presence of several additional compounds that do not belong to the classes mentioned above. The presence of choline (Cho) was showed by the characteristic methyl singlet resonating at δ 3.15 and confirmed by comparison with pure standard (Figure 4b). In addition, the presence of ethanolamine (Eta) was demonstrated by its characteristic double triplet resonating at δ 3.15 (β-CH_2_, *J* = 6.8, 4.0) (Figure 4b). The phenolic acid chicoric acid (Chi) was identified by the proton signals of the trans double bond resonating as coupled doublets at δ 6.46 and 7.70 (each d, *J* = 16 Hz) (Figure 4c and Table 2). The same functional group allowed the identification of chlorogenic acid (Chl) with the protons of the trans double bond resonating at δ 6.40 and 7.62 (each d, *J* = 16 Hz). Lastly, trigonelline (Tri) was identified by the proton signals at δ 8.05 (CH, t), 8.80 (CH, t) and 9.09 (CH, s) (Figure 4c and Table 2).

### 2.3. Chemometric Analysis

A multivariate analysis was performed on the matrix of integrated NMR spectral data reported in supplementary (Figure S1 and Table S1).

**Figure 5:**
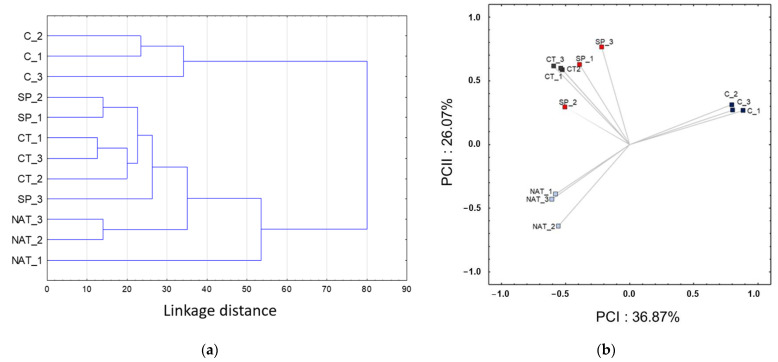
Numerical clustering (**a**) and principal component analysis (PCA) (**b**) of the three replicates of integrated NMR spectral data of control (C) and treated plants (CT, SP and NAT). The classification dendrogram clearly aggregates the control group separately from all fertilization treatments. The ordination plot also shows a clear separation along the first principal component of the C versus treated plants, whereas the second component segregates the NAT from the other organic fertilization treatments.

Firstly, the dendrogram produced by the numerical clustering (Figure 5a) shows a strong separation between the group of control plants and the general cluster aggregating all treated plants. Moreover, in the latter group, the three different treatments maintained a clear segregation with sub-groups of plants irrigated with compost-tea CT) aggregated together and joining to those treated with spirulina (SP) with high similarity (linkage distance < 26). The NAT treatment showed a lower level of homogeneity, with their cluster grouping with all the others at a lower similarity level (linkage distance > 50).

Secondly, the ordination plot obtained by PCA (Figure 5b) was highly informative regarding the similarity and trends of variation of the spectral data of plants in the different fertilization treatments. The first principal components, accounting for over 36% of the total variability, clearly separated the control (C) from all organic fertilization treatments, whereas the second axis, still highly relevant because accounting for 26% of variability, showed an aggregation of CT and SP separated by the NAT group. The complete PCA plots including the variable vectors are reported in Figure S5.)

### 2.4. Metabolomic Treatment Comparison

**Figure 6:**
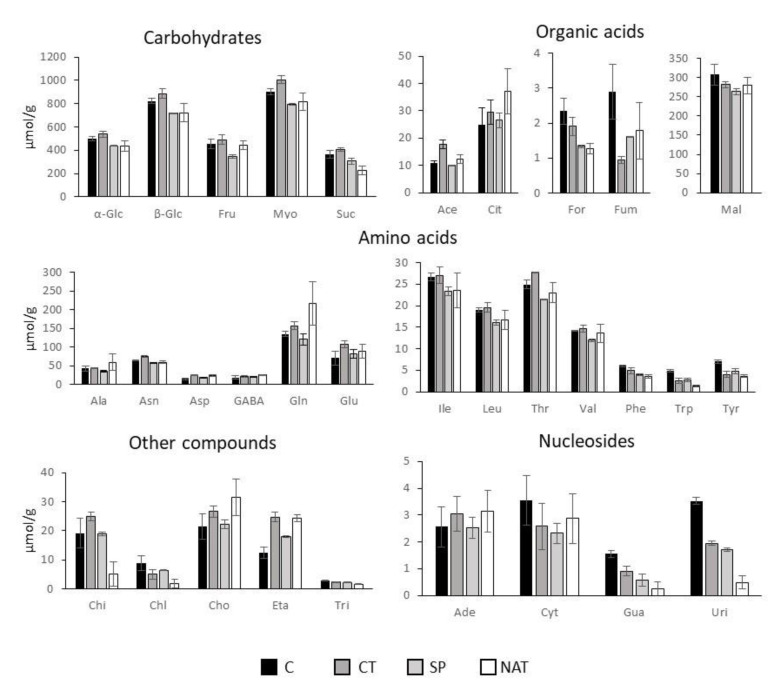
Absolute abundance (µmol/g dry leaves) of the metabolites identified by ^1^H NMR in *L. sativa* leaves extracts. Displayed data refers to the mean and standard deviation of three replicates. The vertical bars represent the four treatments: C = control; CT = compost T, SP = spirulina, NAT = spirulina + *Fusarium* DNA.

A general comparison of the three different treatments with the control plants showed the same number of metabolites in the analyzed samples with differences related to their quantity. Thus, no marker metabolites were observed linked with specific treatments. The integration of the ^1^H NMR spectra allowed acquisition of quantitative data of the identified metabolites (Table S1) and evaluation of their change in the analyzed samples (Figure 6).

The treatment with CT produced an increase in all the identified carbohydrates compared to control plants. The same increase was observed for acetic and for citric acids. The other organic acids were significantly decreased by CT treatment, especially fumaric acid that decreased its concentration by about 67% compared to C. For amino acids a general trend was observed, with all of them slightly increasing, the increase being higher for glutamic acid (+35% compared to C). The only exceptions were the aromatic amino acids, that showed a slightly decreasing trend. Nucleosides mostly decreased, while for other compounds an increasing trend was observed for the phenolic acid chicoric acid, and for choline and ethanolamine, the latter doubling its quantity, in contrast to chlorogenic acid that almost halved.

Treatment with SP had the opposite effect on carbohydrates, decreasing their quantity overall, compared to the control. Fumaric and formic acids showed a large decrease after SP treatment. Malic acid only slightly decreased, while the other organic acids remained constant. Regarding amino acids, they were not particularly influenced, all of them remaining almost constant except threonine, tryptophan and tyrosine that showed a slightly decreasing trend. Moreover, SP treatment influenced nucleoside content with a large decrease in cytidine, uridine and guanosine. In detail, guanosine decreased by 62.5% and uridine by 51%, both compared to C. Among other compounds, ethanolamine showed an increasing trend, while chlorogenic acid slightly decreased.

NAT treatment resulted in a slight modification of carbohydrate content, with a slight decrease in sucrose, but influenced organic acid content to a greater extent, especially citric acid, that increased (+33%), and formic and fumaric acids, both of which almost halved (−54% and −38%). The only amino acid that dramatically increased after NAT treatment was glutamine (+ 38% compared to C). However, glutamic and aspartic acids, alanine and GABA slightly increased also, while isoleucine, phenylalanine, tryptophan and tyrosine showed a decreasing trend. Going into detail, tryptophan decreased by 73% and tyrosine by 48%, the others showing a minor decrease. Regarding nucleosides, NAT treatment only caused decreased nucleosides, with uridine showing the largest compared to C (86%). Moreover, decreasing trends were observed for chicoric and chlorogenic acids, while ethanolamine and choline increased by 32% and 48%, respectively, compared to control plants.

## 3. Discussion

To our knowledge, this is the first report of the characterization of the metabolome of lettuce leaves by NMR metabolomics under organic foliar fertilization treatment, studied under field conditions. Comparison of the three different organic foliar fertilization treatments and the control plants have been performed by using a fast and efficient protocol previously validated in other metabolomics analyses of food plants [30,31].

Regarding the agronomic results, it should be noted that the control plants in this study were cultivated in a soil loaded with the fungal pathogen FOL and showed a significant occurrence of the disease during the crop cycle. In the control plots about 20% of the lettuce plants died and a similar number of plants reported stunted growth. All fertilization treatments, especially NAT, reduced the occurrence of the fungal disease in terms of decreasing numbers of both dead and stunted plants. Moreover, the average weight of individual lettuces increased with all fertilization treatments. Among these, NAT again performed better.

Regarding the metabolomic study, several primary and secondary metabolites were identified and quantified in the organic extracts of the analyzed samples by using NMR metabolomics. The data agree with previous studies reporting the chemical composition of lettuce [7,8,9,12], thus confirming the method used as an efficient and valid protocol for analysis. The data obtained, indicate that fertilization treatments did not significantly affect the general plant metabolite composition. However, evident metabolite trends were observed and some agreed with previous studies.

Carbohydrates were the main compounds in the extracts. They showed a slight increase with CT treatment while the microalgae (both SP and NAT) produced a detectable decreasing trend. According to Zhou et al. [10], stress conditions may increase sugar production, thus we can relate the effect of NAT and SP with the known biostimulation reported for spirulina. An increase from stress was also observed by the same authors for the aromatic amino acids Tyr and Trp, while a decrease was observed for the amino acids, Glu and Asp. Our data totally agree with this finding, showing higher levels of Tyr, Trp and Phe in the control plant, while lower levels were observed for Glu and Asp.

Similar effects of stress conditions were found by Wei et al. [11] for the amino acids Val, Ile and Thr. Our data also agrees with their findings, demonstrating an improvement of the physiological status of lettuce plants after fertilization. Concerning the organic acids, as recently reported by Matamoros et al. [12], increased levels of organic acids, mainly fumaric acid, are triggered by stressful conditions or lack of nutrients with involvement of the TCA cycle and related energy production. Our findings of reduced amounts of the organic acids Fum, For and Mal after fertilization treatment confirm the healthiness of the fertilized plants compared to the untreated control plant.

The reported results are interesting because on one hand, as expected, they confirmed that plants treated by organic foliar fertilization do have a physiological response reflected by variations in their metabolome. Our observations confirmed other reported published effects [7,8,9,10,11,12], showing variations of specific metabolites reflecting a reduction in either biotic or abiotic stresses compared to control. On the other hand, results were very surprising when showing a higher similarity between SP and CT *versus* NAT treatment. In fact, considering that both SP and NAT treatments were based on lyophilized pellets of the same microalgal species (*A. platensis*) it was reasonably expected that SP and NAT would have shared a higher similarity in their effects on the treated lettuce plants. On the contrary, exposure to FOL DNA in the preparatory steps of the natural microbial library (NAT), evidently induced a variation in the spirulina biochemical characteristics that were somehow stabilized and carried over, during the scale up process, up to producing different effects and metabolic reactions in the treated plants.

Cyanobacteria are well known for their high transformation capability [32]. This seemed to be confirmed by the fungal DNA observed effects, but the modes of action and the mechanisms of the possible integration in the microalgal genome, remain to be investigated in further work.

In this work, the control plants were affected by FOL, i.e., the same fungal pathogen whose self-DNA was used in the preparation of the natural microalgae of the NAT treatment. So, our results, although being produced by a still preliminary study, represent a first proof of concept of the biocontrol idea based on the use of self-DNA of a pathogen species, as proposed by Mazzoleni et al. [27,28]. In other words, while the general biostimulation by organic fertilization may act as a strengthening of the treated plants, some additional beneficial effects seem to be associated with the combined effect of plant biostimulation with a pathogen self-DNA inhibition.

## 4. Materials and Methods

### 4.1. Field Site and Experimental Design

The experiment was performed at the farm “Azienda De Vita Rosario” located in Battipaglia, Campania Region, Italy (40°34′39.6″ N 14°58′27.5″ E). The site is located in an alluvial plain with highly fertile soils extensively used for horticulture in both open field cultivation and greenhouses. The climate is typical Mediterranean with rains concentrating in winter and usually above 1000 mm/year and mild average temperature, almost never freezing in winter and steadily above 30 °C in summer. Light insolation is comparable to optimal conditions as reported for lettuce cultivation [33].

The lettuce plants were provided by the nursery of Citro Giuseppe, associated with the farm “Finagricola”. The *Lactuca sativa* cultivar was selected as being not resistant to *Fusarium oxysporum lactucae* (FOL).

The experiment was organized according to a randomized block design with three blocks including 100 individual plants each in 5 rows (three central and two laterals in each block). Three different treatments of foliar application included:

(1) CT: a commercial compost tea named Stimol-C^®^ produced by GWA—Gima Water & Air S.r.l. and made of mixed plant materials and cow manure from biological farms. The physical and chemical characteristics before starting the aerobic fermentation are the following: humidity 13%, pH: 6.8, organic C: 27%, humic and fulvic acids: 8.2%, organic N: 2.3%, C/N ratio: 11.7, salinity: 12.4 dS/m.
(2) SP: *Artrospira platensis* (spirulina) selected strain by M2M Engineering, cultivated in a photobioreactor to produce the organic pellet lyophilized and used in the foliar application treatment.
(3) NAT: The same strain of *A. platensis* used in the SP treatment was exposed in its preparation to a natural uptake from a culture medium enriched with *Fusarium oxysporum* fragmented DNA before its scale-up in the photobioreactor to produce the organic pellet used in the foliar application treatment.

### 4.2. Fusarium Oxysporum Lactucae (FOL) Phytopathogenic Analyses

Plants with wilting symptoms, collected from different parcels, were transported to the laboratory and examined. Small fragments were aseptically excised from discolored vascular tissues (see Figure 1 bottom right inset) placed on Petri dishes of Difco potato dextrose agar, with lactic acid added or not (0.7 mL of 25% lactic acid for 100 mL of PDA medium). Plates were incubated at 24 °C and analyzed after one week. Fungal colonies with white abundant aerial mycelium (see Figure 1 bottom left) showing a purple–red color on the reverse side of the plates, were observed. Typical micro- and macro-conidia seen at 400 × optical microscope magnifications allowed the identification of the colonies as *F. oxysporum* in all the cases that were examined.

### 4.3. Natural Microbial Library Preparation

The *F. oxysporum* mycelium was grown in pure culture in petri dishes and flasks. The mycelium was ground in liquid nitrogen and then suspended in a double volume of SDS buffer at pH 8.0. The sample was centrifuged and aerobically decomposed in highly controlled conditions to increase the DNA concentration. The DNA concentration and fragmentation level in the samples were checked using standard extraction methods and evaluated by QUBIT (Thermo Fisher, Third Avenue Waltham, MA, USA) fluorimeter and electrophoresis in 1% agarose gel. The samples were sheared using a Bioruptor Plus (Diagenode, Seraing (Ougrée) Belgium, EU) sonicator and then lyophilized.

The production of natural libraries to obtain biomass for trials (NAT treatment), comprised several upscaling steps, from small volume flasks in the laboratory in climatic chambers in controlled conditions, to medium scale photobioreactors in controlled conditions for continuous production. The cultures were grown in the first steps of production in small laboratory scale in flasks in climatic chamber in controlled conditions of temperature (25 °C) with irradiation intensity 200 μmol/m^2^ s and a photoperiod cycle of 12 h day/12 h night, with cells grown in medium substrate containing random DNA fragmented in classic normal medium. The growth medium used was the classic Zarrouk medium for *A. platensis*. Cultures were grown and scaled-up in still 2 L flasks in a climatic chamber and after in the small-scale photobioreactor M2M-PBR-10, with a volume of 10 L in the laboratory. The conditions in the photobioreactor were fully controlled and optimized, with an optimized LED lighting spectrum, with the temperature of the culture at 25 °C, lighting 12 h day/12 h night, irradiation 400 μmol/m^2^ s. The cultures were then scaled-up in two medium scale photobioreactors (M2M-PBR-150), with culture volumes of 150 L, irradiance 400 μmol/m^2^ s 12 h day/12 h night LD. Photobioreactors are optimized and controlled for continuous and fully controlled production of microalgae with LED lighting spectra optimized for cultivation. Biomass is continuously produced in the photobioreactor, and periodically harvested, dried, and stored in closed packaging for application in field trials. The process for production of microalgal biomass for the basic foliar fertilization treatment (SP) was identical to that described above for the NAT protocol, with the same and parallel steps of scaled-up growing volumes and systems used: flasks, photobioreactors, lab scale, and photobioreactor medium scale. Timing, harvested amounts, and preparation of dried pellets to be used in the foliar application were the same.

### 4.4. Data Pre-Processing

Data from ^1^H NMR (nuclear magnetic resonance) were preprocessed before statistical analysis. In detail, the spectral region was first normalized to total area, to minimize small differences and subsequently, mean centered applied to minimize the problem of heteroskedasticity in the data. Further, because of the high dominance of most abundant peaks, data were pareto scaled. The process of pretreatment was applied for both polar and apolar data.

### 4.5. Multivariate Data Analysis

Data from NMR spectra were analyzed using multivariate statistical procedures. Prior to data transformation cluster analyses were run to observe the similarity among sample groups, according also to quantitative information of metabolites within each spectrum. Cluster analyses were made by creating a contingency matrix based on Euclidean distance and a successive hierarchical dendrogram built based on complete linkage methodology. After data normalization and transformation both polar and apolar spectra were analyzed by principal component analysis (PCA) to examine the intrinsic variation in the dataset, and specifically, in metabolic composition of the sample material. Moreover, the methodology allows observation of specific associations between sample groups and metabolite signals according to graphical disposition in multidimensional space. Both cluster analysis and dendrograms, and PCA, were performed by means of Statistica 10 software (StatSoft: Tulsa, OK, USA).

### 4.6. Chemicals

First-grade dichloromethane and methanol were purchased from Delchimica Scientific Laboratories Glassware (Naples, Italy). Deuterium oxide (99.8 atom %D) and dimethyl-4-silapentane sodium sulfonate (DSS) was obtained from ARMAR Chemicals (Döttingen, Switzerland), chloroform-d (99.8 atom %D) containing 0.03% (*v*/*v*) TMS was purchased from Sigma-Aldrich (Milan, Italy). Pure standard amino acids, chlorogenic acid, and nucleosides were used as references (Sigma-Aldrich, Milan, Italy).

### 4.7. Metabolite Extraction Procedure

Young lettuce leaves (20 g) were harvested from each treatment plot after 30 days since the experiment start and immediately frozen at −80 °C. The leaves were then freeze-dried by lyophilization for two days and ground in a mortar, obtaining a fine green powder. One hundred milligrams of each powdered sample were collected in Eppendorf tubes and 1 L of dichloromethane was added to each tube to perform a non-polar extraction. The mixtures were sonicated for 10 min and then centrifuged at 7000 rpm for 10 min, RT. The supernatants were collected into 10 L vials and dried at room temperature. The remaining pellets were dried at room temperature for 2 h, then a polar extraction was performed, by adding 1 L of a methanol/water solution (1:1 *v*/*v*) to each tube. The mixtures were sonicated again for 10 min, and then centrifuged at 7000 rpm for 10 min, RT. The supernatants were collected into new Eppendorf tubes and dried using a speed vac. These dried extracts were kept at −20 °C until NMR analysis was performed.

### 4.8. NMR Metabolomic Analysis

Dried aqueous fractions were diluted in 600 μL of D_2_O (99.8%), while dried organic fractions were dissolved in 600μL of CDCl_3_ (99.8%) and transferred into 5 mm NMR tubes. DSS and TMS, both 0.03% (*v*/*v*) in D_2_O and CDCl_3_, respectively, were used as internal standards for aqueous and organic fractions, respectively. The pH of the aqueous fractions was adjusted to 6.0 by using potassium dihydrogen phosphate (KH_2_PO_4_) as a buffering agent and 1 N sodium deuteroxide (NaOD). The NMR spectra were recorded at 298 K with a Varian Unity Inova spectrometer operating at 600 MHz. For each sample 200 transients were recorded using a spectral width of 12 ppm on 32 K data points and a relaxation delay of 0.04 s. Chemical shifts were referred to DSS and TMS signals (both 0.00 ppm). All spectra were processed using the iNMR program (www.inmr.net), phased and baseline corrected manually. Quantification was performed by signal integration relative to the internal standard, DSS and TMS. The region of the solvent peaks was excluded from the analysis. Spectral peak assignments of organic acids, amino acids, carbohydrates, chlorogenic acid and its derivatives were obtained based on pure standards purchased from Sigma-Aldrich and interpretation of 2D NMR experiments. Spectral peak assignments of these and the other detected metabolites were obtained by two-dimensional (2D) NMR experiments, including ^1^H-^1^H correlation spectroscopy (COSY) and ^1^H-^13^C heteronuclear single-quantum correlation (HSQC) and comparison with data reported in the literature. The COSY spectra were acquired with a spectral width of 6130 Hz in both dimensions, 8 K data points, and 512 increments with 32 transients per increment. The HSQC spectra were acquired with spectral widths of 8000 Hz in the F2 dimension and 25,000 Hz in the F1 dimension, a data matrix with a size of 1 K × 256 data points, and 64 transients per increment. The obtained values showed a very good repeatability, with coefficient of variation among replicates < 2.5% for all signals.

## 5. Conclusions

Our findings suggest the possibility of many further studies, firstly to confirm the reported observations, but also to investigate the mechanism of uptake and incorporation of the fungal DNA in the microalgae and its potential optimized use for pathogen control. Further experimental work will have to focus on better definition of the administration protocol and dosages of the foliar application treatments. Likely, the anticipation of the treatments in earlier phases of the plantation will enhance the beneficial effects, reinforcing plant resistance to the pathogen infection and thus better inhibiting disease development.

## 6. Patent

WO2014020624—Composition comprising nucleic acids of parasitic, pathogenic or weed biological systems for inhibiting and/or controlling the growth of said systems.

## Figures and Tables

**Table 1:** Number of plants either dead or with reduced growth because of *Fusarium oxysporum* infection and weight of individuals in control plots (C) and in different foliar application treatments with compost tea (CT), spirulina (SP), and spirulina previously exposed to natural uptake of *Fusarium* DNA (NAT). Weight data are averages of random 10 plants and corresponding standard deviations.

Treatment	Dead Plants (#)		Stunting Plants (#)		Weight (g)	
	AVG	SD	AVG	SD	AVG	SD
**C**	18.67	5.51	17.00	5.29	377.40	67.87
**NAT**	6.00	2.65	10.33	7.51	493.60	73.80
**SP**	7.33	1.15	13.67	4.16	411.60	103.47
**CT**	6.67	2.08	17.67	3.06	487.50	106.66

**Table 2:** ^1^H NMR chemical shifts, assignment, and multiplicity at 600 MHz in D_2_O of the metabolites detected in all analyzed polar extracts of *L. sativa* plants.

Compound	Assignment	^1^H (ppm)	Multiplicity [J (Hz)]
*Organic acids*			
Acetic acid (Ace)	α-CH_3_	1.88 *	s
Citric acid (Cit)	CH_2_	2.59 *	dd [15.0, 15.0]
Formic acid (For)	COOH	8.41 *	s
Fumaric acid (Fum)	α-CH	6.48 *	s
Malic acid (Mal)	β’-CH_2_	2.36	dd [15.7, 8.9]
	β-CH	2.65	dd [15.7, 3.7]
	α-CH	4.28 *	dd [8.9, 3.7]
*Amino acids*			
Alanine (Ala)	β-CH_3_	1.43 *	d [7.0]
Asparagine (Asn)	β-CH	2.86	dd [17.4, 3.8]
	β-CH	2.87 *	dd [16.9, 7.3]
Aspartic acid (Asp)	β-CH_2_	2.76 *	dd [17.4, 3.8]
γ-aminobutyrate (GABA)	β-CH_2_	1.87	m
	α-CH_2_	2.26	t [7.0]
	γ-CH_2_	2.97 *	t [7.0]
Glutamic acid (Glu)	β-CH_2_	2.02	m
	γ-CH_2_	2.32 *	m [7.0]
Glutamine (Gln)	β-CH_2_	2.10	m
	γ-CH_2_	2.43 *	m
Isoleucine (Ile)	δ-CH_3_	0.91 *	t [7.0]
	γ’-CH_3_	1.00	d [7.0]
Leucine (Leu)	δ-CH_3_	0.95 *	d [7.0]
Phenylalanine (Phe)	CH-2,6	7.28	d [7.0]
	CH-4	7.33	t [7.0]
	CH-3,5	7.38 *	d [7.0]
Threonine (Thr)	γ-CH_3_	1.29 *	d [6.4]
Tryptophan (Trp)	CH-6	7.18	d [7.5]
	CH-7	7.49 *	d [7.5]
	CH-4	7.69	d [7.5]
Tyrosine (Tyr)	CH-3,5	6.85 *	d [7.0]
	CH-2,6	7.14	d [7.0]
Valine (Val)	γ’-CH_3_	0.97	d [7.0]
	γ-CH_3_	1.00 *	d [7.0]
*Carbohydrates*			
α-Glucose (α-Glc)	CH-1	5.19 *	d [4.0]
β-Glucose (β-Glc)	CH-1	4.61 *	d [8.0]
Fructose (Fru)	CH-4	3.90	dd
	CH_2_-6	4.00 *	dd
Myo-inositol (Myo)	CH-4	3.20 *	t [9.50]
Sucrose (Suc)	Glc CH-1	5.38 *	d [4.0]
	Fru CH-3	4.18	d [8.5]
*Nucleotides*			
Adenosine (Ade)	CH-2	8.21 *	s
	CH-8	8.40	s
Cytidine (Cyt)	CH-1^I^	6.03	d [3.5]
	CH-6	7.82 *	d [7.0]
Guanosine (Gua)	CH-8	7.95 *	s
Uridine (Uri)	CH-6	7.91 *	d [7.0]
*Other compounds*			
Chicoric acid (Chi)	CH-2	6.46 *	d [16.0]
	CH-3	7.70	d [16.0]
Chlorogenic acid (Chl)	CH-2	6.40 *	d [16.0]
	CH-2^I^	7.10	dd [8.2, 2.2]
	CH-3	7.62	d [16.0]
Choline (Cho)	N(CH_3_)^3+^	3.15 *	s
Ethanolamine (Eta)	β-CH_2_	3.10 *	dt [6.8, 4.0]
Trigonelline (Tri)	CH	8.05	t
	CH	8.80 *	t
	CH	9.09	s

* Signal used for quantitation.

## Data Availability

Not applicable.

## Supplementary Materials

The following supporting information can be downloaded at: https://www.mdpi.com/article/10.3390/plants11162164/s1, Figure S1: ^1^H NMR spectra; Figure S2: 2D COSY; Figure S3: 2D HSQC; Figure S4: 2D HSQC; Figure S5: Principal Component Analysis; Table S1: Quantitative data of the detected metabolites.
