# Supplementary material for: NMR Metabolomics and Chemometrics of Lettuce, Lactuca sativa L., under Different Foliar Organic Fertilization Treatments
Source: Plants (Basel). 2022 Aug 20;11(16):2164. doi: 10.3390/plants11162164 (PMC9413100; doi:10.3390/plants11162164)
Supplement: Supplementary file 1 [file plants-11-02164-s001.zip › plants-1850484-supplementary.pdf]

Article

NMR Metabolomics and Chemometrics of Lettuce, *Lactuca sativa* L., under Different Foliar Organic Fertilization Treatments

Virginia Lanzotti [1,*], Attilio Anzano [1], Laura Grauso [1], Maurizio Zotti [1], Adriana Sacco [2], Mauro Senatore [2], Mauro Moreno [1], Marcello Diano [3], Maddalena Parente [3], Serena Esposito [3], Pasquale Termolino [4], Emanuela Palomba [4], Astolfo Zoina [1] and Stefano Mazzoleni [1,*]

1 Dipartimento di Agraria, Università di Napoli Federico II, Via Università 100, 80055 Portici, Italy
2 Institute for Sustainable Plant Protection (IPSP), National Research Council of Italy (CNR), 80055 Portici, Italy
3 M2M Engineering sas, Business Innovation Center, Science Center, Via Coroglio, 80124 Naples, Italy
4 Institute of Biosciences and Bioresources (IBBR), National Research Council of Italy (CNR), 80055 Portici, Italy
* Correspondence: virginia.lanzotti@unina.it (V.L.); stefano.mazzoleni@unina.it (S.M.); Tel.: +39-0812539459 (V.L.); +39-0812532020 (S.M.)

SUPPLEMENTARY MATERIAL

Figure S1. ^{1}H NMR spectra in triplicates of the *Lactuca sativa* leaves extracts treated with commercial compost tea (CT), Spirulina (SP), and Spirulina + *Fusarium* DNA (NAT). C indicates control plants. Spectra were registered in D_2O at 600 MHz.

Figure S2. 2D COSY of the control plant registered in D_2O at 600 MHz.

Figure S3. 2D HSQC of the control plant registered in D_2O at 600 MHz.

Figure S4. 2D HSQC of the control plant registered in D_2O at 600 MHz.

Figure S5. Principal Component Analysis (PCA) plots of first and second axis of all treatments and corresponding NMR spectral signals.

Table S1. Quantitative data (µmol/g dried leaves) of the detected metabolites in the analysed samples. AVG= average; SD= standard deviation

Figure 1. ^{1}H NMR spectra in triplicates of the *Lactuca sativa* leaves extracts treated with commercial compost tea (CT), Spirulina (SP), and Spirulina + *Fusarium* DNA (NAT). C indicates control plants. Spectra were registered in D_2O at 600 MHz.

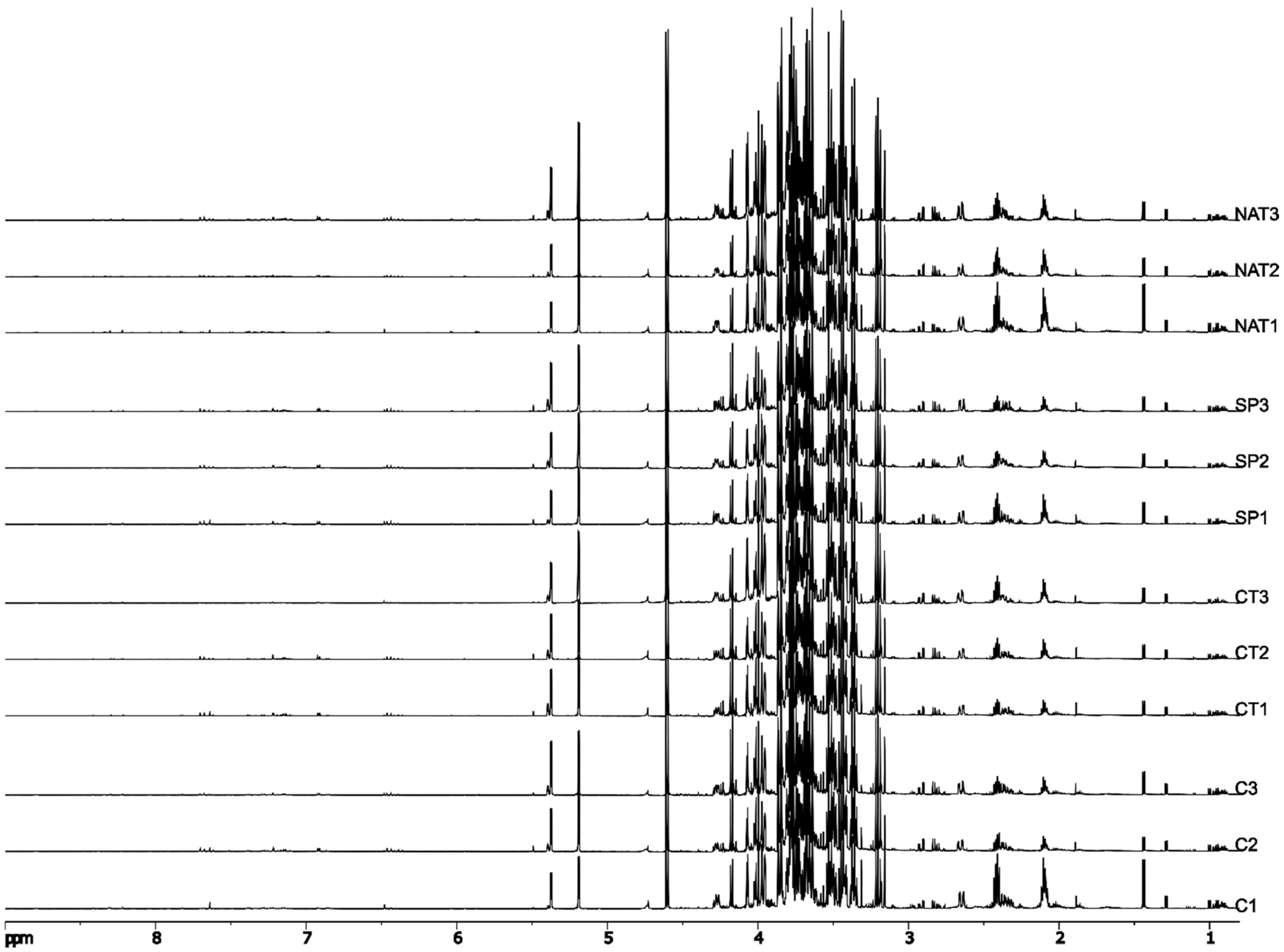

Figure S2. 2D COSY of the control plant registered in D_2O at 600 MHz.

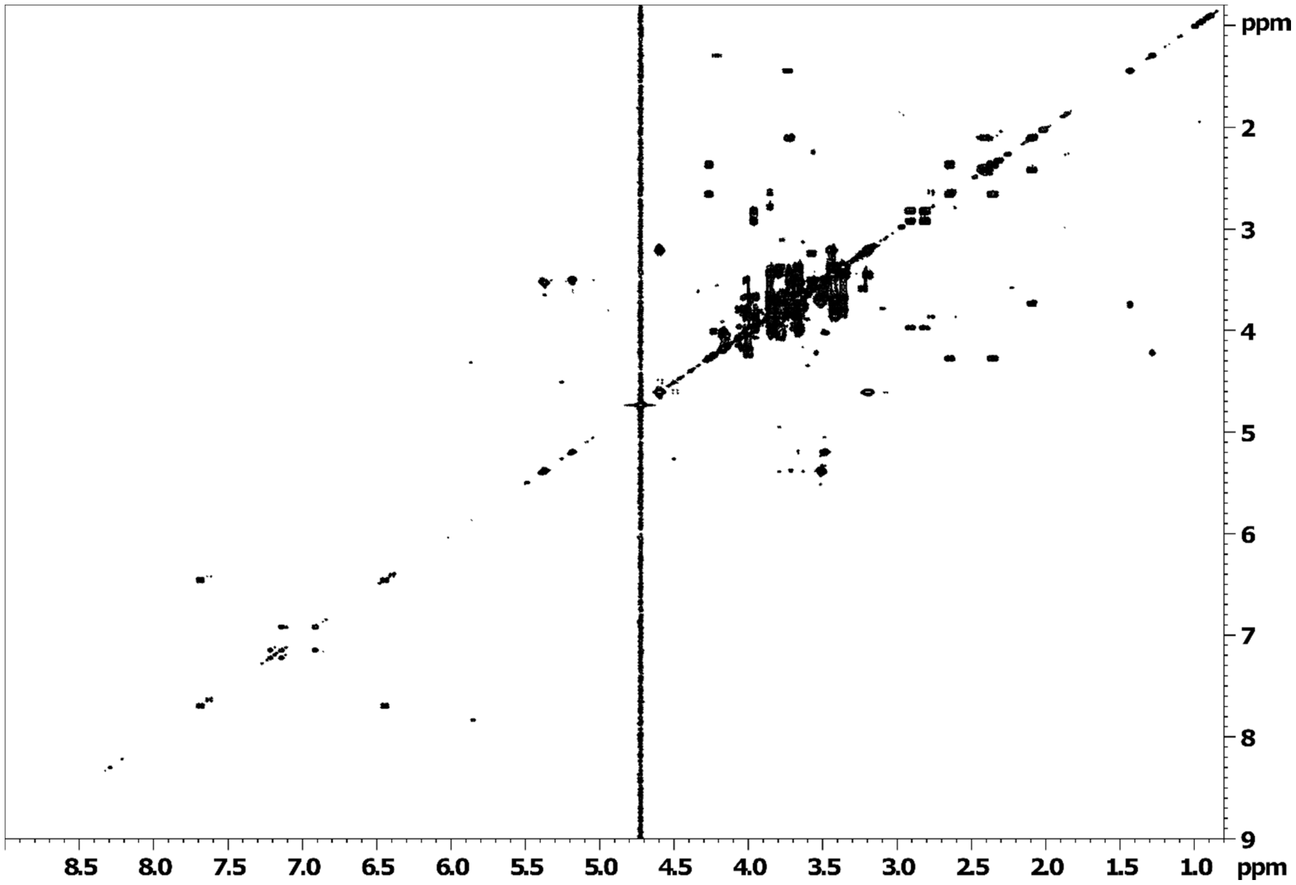

Figure S3. 2D HSQC of the control plant registered in D_2O at 600 MHz.

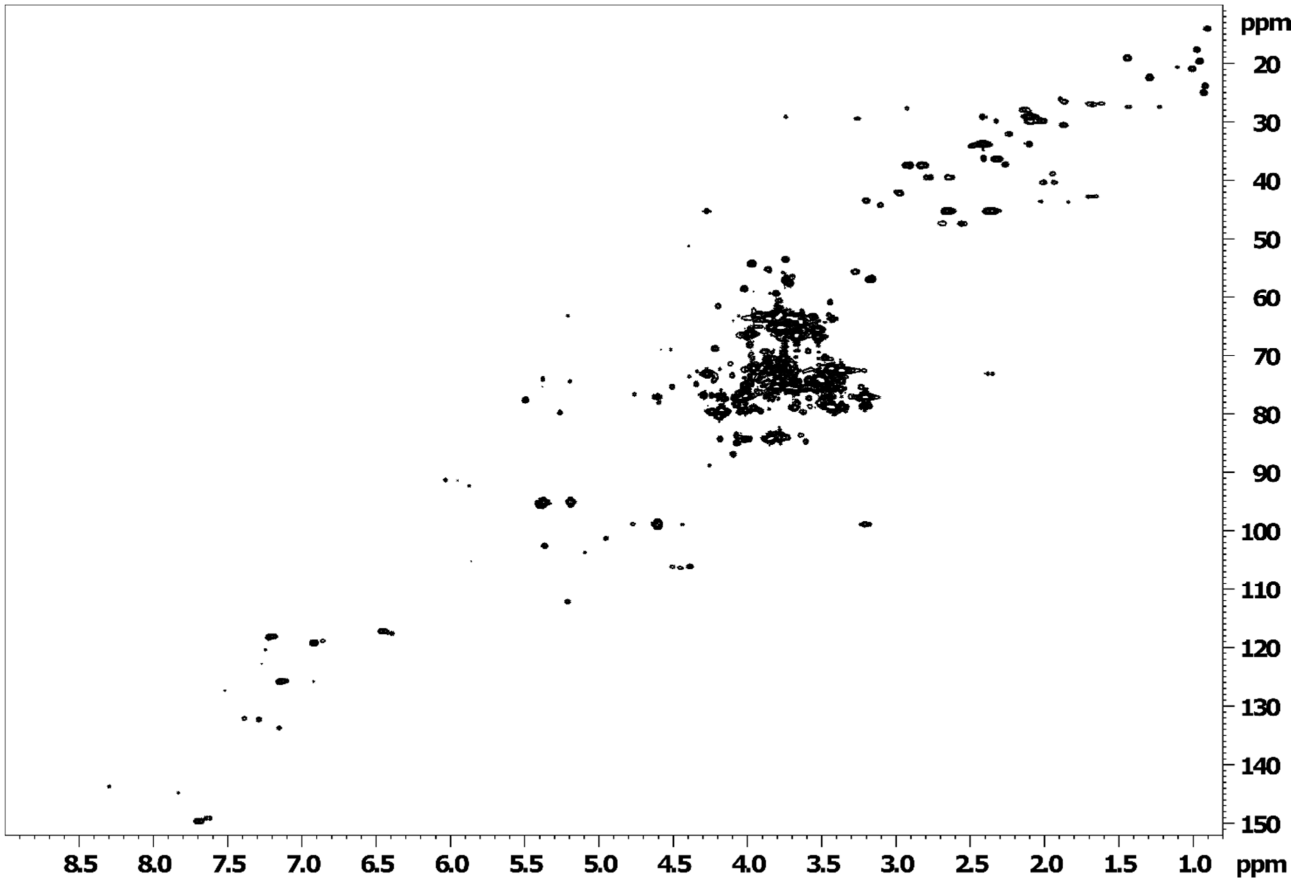

Figure S4. 2D HMBC of the control plant registered in D_2O at 600 MHz.

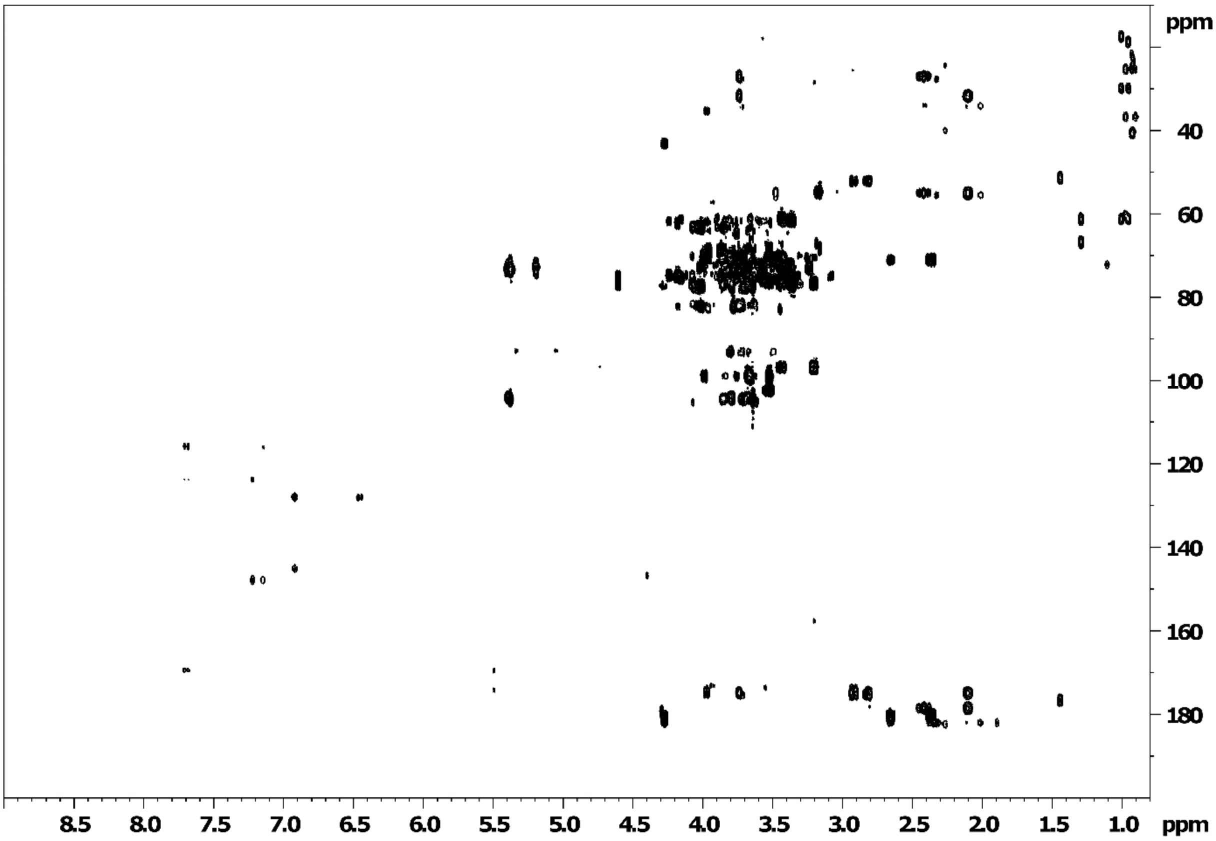

Figure S5. Principal Component Analysis (PCA) plots of first and second axis of all treatments and corresponding NMR spectral signals.

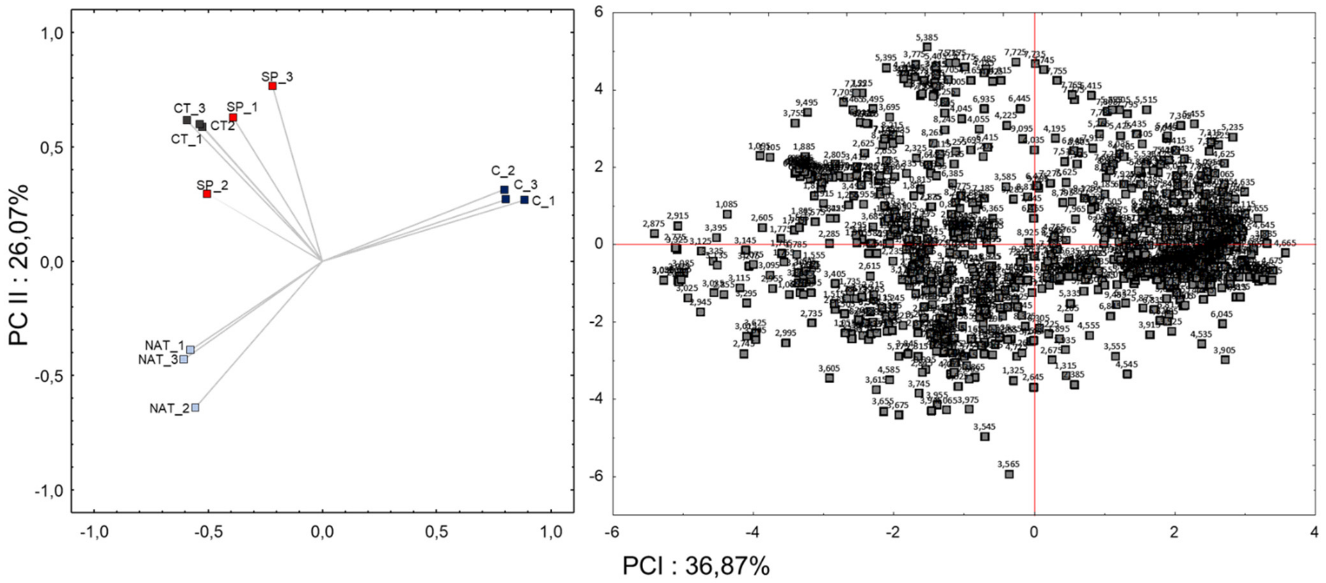

Table S1. Quantitative data (µmol/g dried leaves) of the detected metabolites in the analysed samples. AVG= average; SD= standard deviation

	**C**	**CT**	**SP**	**NAT**
**Compound**	**AVG±SD**	**AVG±SD**	**AVG±SD**	**AVG±SD**
Organic acids				
Acetic acid	10.94±0.90	17.75±1.59	10.06±0.02	12.30±1.65
Citric Acid	24.89±6.31	29.58±4.52	26.55±2.69	37.18±8.16
Formic acid	2.34±0.37	1.91±0.26	1.34±0.04	1.27±0.15
Fumaric Acid	2.90±0.79	0.95±0.10	1.61±0.02	1.79±0.81
Malic acid	307.48±26.51	281.75±8.47	263.10±6.87	279.13±20.90
Amino acids				
Alanine	42.85±7.45	43.92±1.15	35.38±1.69	59.32±21.63
Asparagine	64.41±0.90	74.77±3.33	57.59±0.44	59.10±3.57
Aspartic acid	15.42±2.12	24.66±1.67	18.96±1.29	23.35±1.94
GABA	18.62±4.10	21.69±1.70	20.48±1.03	25.15±1.78
Glutamic acid	70.58±17.91	107.06±9.76	81.79±11.64	87.82±18.61
Glutamine	134.70±6.92	157.13±11.21	120.22±14.76	216.60±57.79
Isoleucine	26.61±0.87	27.06±1.96	23.39±0.97	23.56±4.07
Leucine	18.94±0.48	19.53±1.13	16.12±0.66	16.69±2.29
Phenylalanine	5.99±0.19	4.87±0.78	3.97±0.15	3.56±0.35
Threonine	24.86±1.01	27.82±0.07	21.58±0.01	23.02±2.33
Tryptophan	4.91±0.30	2.50±0.56	2.81±0.39	1.32±0.29
Tyrosine	6.99±0.43	3.95±0.71	4.77±0.68	3.63±0.37
Valine	14.25±0.03	14.70±0.78	11.96±0.34	13.55±2.18
Carbohydrates				
α-Glucose	499.68±15.12	538.54±24.02	438.73±1.55	437.76±44.95
β-Glucose	822.37±23.84	887.30±38.88	721.72±0.94	721.22±78.10
Fructose	455.43±38.89	490.97±40.03	345.82±17.26	444.27±38.10
Myo-Inositol	903.42±25.24	1006.79±32.33	796.19±7.07	814.37±75.36
Sucrose	364.62±32.72	407.41±17.00	306.03±25.06	228.88±38.64
Nucleosides				
Adenosine	2.56±0.76	3.04±0.65	2.52±0.39	3.14±0.77
Cytidine	3.56±0.92	2.58±0.87	2.31±0.37	2.87±0.93
Guanosine	1.55±0.12	0.91±0.17	0.58±0.23	0.25±0.26
Uracil	3.53±0.13	1.95±0.08	1.71±0.06	0.48±0.24
Other compounds				
Chicoric Acid	19.12±5.09	24.84±1.42	18.82±0.56	5.07±4.14
Chlorogenic acid	8.89±2.54	5.01±1.64	6.48±0.25	1.88±1.54
Choline	21.36±4.35	26.67±1.97	22.22±1.55	31.59±6.30
Ethanolamine	12.46±2.03	24.73±1.72	17.96±0.26	24.23±1.16
Trigonelline	2.92±0.20	2.38±0.04	2.23±0.23	1.65±0.14
